# Genomic homeology between *Pennisetum purpureum* and *Pennisetum glaucum* (Poaceae)

**DOI:** 10.3897/CompCytogen.v8i3.7732

**Published:** 2014-08-08

**Authors:** Gabriela Barreto dos Reis, Amanda Teixeira Mesquita, Giovana Augusta Torres, Larissa Fonseca Andrade-Vieira, Antônio Vander Pereira, Lisete Chamma Davide

**Affiliations:** 1 Departament of Biology, Federal University of Lavras, Zip Code 37200-000, Lavras, MG, Brazil; 2 Laboratory of Plant Genetics, Brazilian Agricultural Research Corporation, Embrapa Dairy Cattle, Zip Code 36038-330, Juiz de Fora, MG, Brazil

**Keywords:** Homeology, *Pennisetum purpureum*, *Pennisetum glaucum*, Genomic *in situ* hybridization

## Abstract

The genus *Pennisetum* (Richard, 1805) includes two economically important tropical forage plants: *Pennisetum purpureum* (Schumacher, 1827) (elephant grass), with 2n = 4x = 28 chromosomes and genomes A'A'BB, and *Pennisetum glaucum* (Linnaeus, 1753) (pearl millet), with 2n = 2x = 14 chromosomes and genomes AA. The genetic proximity between them allows hybrids to be obtained (2n = 3x = 21) that yield forage of higher quality in relation to the parents. The study of genomic relationships provides subsidies for the knowledge about phylogenetic relations and evolution, and is useful in breeding programs seeking gene introgression. Concerning elephant grass and pearl millet, the homeology between the genomes A and A', and between these and the genome B, has been reported by conventional cytogenetic techniques. The objective of the present study was to demonstrate the degree of homeology between these genomes by means of genomic *in situ* hybridization (GISH). The results confirmed the homeology between the genomes A of pearl millet and A'B of elephant grass, and showed that there are differences in the distribution and proportion of homologous regions after hybridization. Discussion regarding the evolutionary origin of *P. purpureum* and *P. glaucum* was also included.

## Introduction

The genus *Pennisetum* (Richard, 1805) is one of the most important in family Poaceae family. It comprises about 140 species, distributed in five sections (*Penicillaria, Brevivalvula, Gymnothrix, Heterostachya* and *Eu-Pennisetum*) based on morphological characteristics (Stapf and Hubbard 1934). The section *Penicillaria* includes the economically most important species: elephant grass [*Pennisetum purpureum* (Schumacher, 1827)], used as forage, and pearl millet [*Pennisetum glaucum* (Linnaeus, 1753)], used as cereal and forage (Martel et al. 2004).

Molecular analyses based on mitochondrial DNA (Chowdhury and Smith 1988), chloroplast DNA (Renno et al. 2001) and repetitive DNA sequences (Ingham et al. 1993) have revealed significant relationship between the genomes of *Pennisetum glaucum*, *Pennisetum purpureum* and *Pennisteum squamulatum* Fresen., suggesting that these three species may have a common origin (Martel et al. 2004). Among these, the cultivated species *Pennisetum glaucum* and *Pennisetum purpureum* are phylogenetically related, possessing a close resemblance between their genomes, constituting a monophyletic group with recent divergence (Martel et al. 2004). In this sense, *Pennisetum glaucum* is an annual, alogamous, diploid species (2n = 2x = 14, genomes AA), with genome DNA content of 4.72 pg, and constitutes the primary genic pool of this genus. In turn, *Pennisetum purpureum* belongs to the secondary genic group and is a perennial, alogamous, tetraploid species (2n = 4x = 28, genomes A'A'BB), with genome DNA content of 4.60 pg (Martel et al. 1997). Both species have approximately the same DNA content (pg), but differ with regards to the monoploid size genome (basic number, x). *Pennisetum purpureum* is a tetraploid and have about half the DNA content (1.15 pg) of the *Pennisetum glaucum* monoploid genome (2.36 pg). Further, *Pennisetum pupureum* have smaller chromosomes than *Pennisetum glaucum* (Martel et al. 2004). This shows that important chromosome changes may be linked to the evolution and divergence among these species (Andrade-Vieira 2010; Barbosa et al. 2003; Martel et al. 2004; Robert et al. 2011).

In spite of their integrating distinct genic groups and differing as to ploidy level, the genetic proximity between these two species becomes evident when the occurrence of natural hybridization is observed. This sexual compatibility is partial, and results in sterile triploid hybrids (2n = 3x = 21, genome AA’B) (Hanna 1987; Martel et al. 2004; Robert et al. 2011; Techio et al. 2006). Cytologically, the genomic proximity has been demonstrated by meiotic analyses of triploids hybrids obtained in breeding programs. During diakinesis and metaphase I in this hybrid, the formation of seven bivalents is frequently observed, resulting from the pairing among chromosomes of genomes A and A’ of *Pennisetum glaucum* and *Pennisetum purpureum*, respectively, as well as seven univalents of genome B of *Pennisetum purpureum* (Jauhar 1968, 1981; Pantulu 1967; Sree Rangasamy 1972; Techio et al. 2005, 2006). The occurrence, even at low frequency, of trivalents and numbers of bivalents above seven suggests both allo- as well as autosindetic pairing among the genomes A, A’ and B (Sethi et al. 1970; Techio et al. 2005, 2006). These observations on the configurations of bivalents and univalents during meiosis, as well as the morphology of metaphase chromosomes, constitute the pioneering works demonstrating homeology among the genomes A, A’ and B. The obtained results suggest that the genome A of *Pennisetum glaucum* has larger homeology with genome A’ of *Pennisetum purpureum*, whereas the origin of genome B has not been defined (Jauhar 1981; Techio et al. 2005, 2006).

In this sense, despite evidence for a common evolutionary origin between *Pennisetum glaucum* and *Pennisetum purpureum* and the economic importance of these species, there are no studies providing more conclusive data with respect to the homeology among genomes A, A’ and B. Thus, the objective of this work is to describe the proportion and distribution of the homologous regions present in genomes A of *Pennisetum glaucum* and A’B of *Pennisetum purpureum*, by cytomolecular analyses using genomic *in situ* hybridization (GISH).

## Material and methods

### Plant material and genomic DNAs

The evaluations were carried out in mitotic metaphases of the parental *Pennisetum purpureum* (access BAG 65) and *Pennisetum glaucum* (access BN2), and of the triploid hybrid originating from this crossing (BAG 65 × BN2). The plant material and genomic DNAs were provided by the Active Germplasm Bank of Elephant Grass (BAGCE) from EMBRAPA Dairy Cattle (Brazilian Research Institute) and elephant grass breeding program, experimental field José Henrique Bruschi, municipality of Coronel Pacheco, Minas Gerais State, Brazil.

### Chromosome preparation

Roots from seeds or cuttings of BAG 65, BN2 and triploid hybrid accession were collected and pretreated with a 12.5 mg.L^-1^ cycloheximide: 150 mg.L^-1^ 8-hydroxyquinoline solution for 2 h 45 min, at 4 °C, and fixed in ethanol: acetic acid solution (3:1), as proposed by Techio et al. (2002). Fixed root tips were digested with pectinase: cellulase (100U:200U) solution in citrate–phosphate buffer (pH 4.8) for 40 min (*Pennisetum glaucum*) and 3 h 30 min (*Pennisetum purpureum* and interspecific hybrid), at 37 °C, in moist chamber. Slides were prepared as proposed by Dong et al. (2000). A root tip was transferred to a slide and macerated with a drop of ethanol: acetic acid solution (2:1) using a fine-pointed forceps. The slide then was warmed over an alcohol flame. It could called flame-drying method.

### Genomic *in situ* hybridization

Genomic DNAs of *Pennisetum glaucum* and *Pennisetum purpureum* were labeling with biotin-16-dUTP through nick-translation reaction method, thus yielding the genomic probes.

The hybridization technique was carried out according to Jiang et al. (1995). The hybridization mixture [55% formamide (v/v), 10% dextran sulfate (w/v), 2X SSC, pH=7.0, and 2 μL of probe marked with biotin] was denatured at 95 °C, for 8 min. Chromosome preparation was denatured with 70% formamide in 2X SSC (saline sodium citrate) at 85 °C, for 1 min 20 sec (Andrade-Vieira et al. 2013) and hybridized in the mixture at 37 °C for, at least, 16 h in a moist chamber. Detection of the probe marked with biotin was performed with streptavidin conjugated with Alexa Fluor® 488. Chromosomes were counterstained with 1 μg.mL^-1^ 4’,6-diamidino-2-phenylindole (DAPI) Vectashield® antifade solution (Vector Laboratories). The slides were evaluated under an epi-fluorescence Nikon Eclipse E600 microscope. Images of interest were digitized by means of a refrigerated monochromatic Nikon DSQi1MC camera, and processed using the software NIS-Element BR 4.00.03 (Nikon) and Adobe Photoshop CS3.

In order to evaluate the level of homeology between genomes A, A’ and B, the chromosomes of five metaphases from each genome were measured, as well as the proportion occupied by the genomic probe, using the Image Tool 3.0 program. The obtained data were used to create karyograms for comparison of the evaluated genomes.

## Results and discussion

Previous analyses of meiotic pairing in the triploid hybrid have showed that the genomes A and A´ are more related. On the other side, between both and the genome B there are affinity/homeology reduced (Jauhar and Hanna 1998, Techio et al. 2005). In this study, hybridization of genomic probes of *Pennisetum glaucum* and *Pennisetum purpureum* were used for the first time to demonstrate and to confirm homeology among the three genomes. It was evaluated the distribution and proportion of these homeologous regions in the family constituted by the parental *Pennisetum purpureum* (BAG 65) and *Pennisetum glaucum* (BN2), and by the triploid hybrid (BAG 65 × BN2) originating from this crossing.

**Figure 1.**
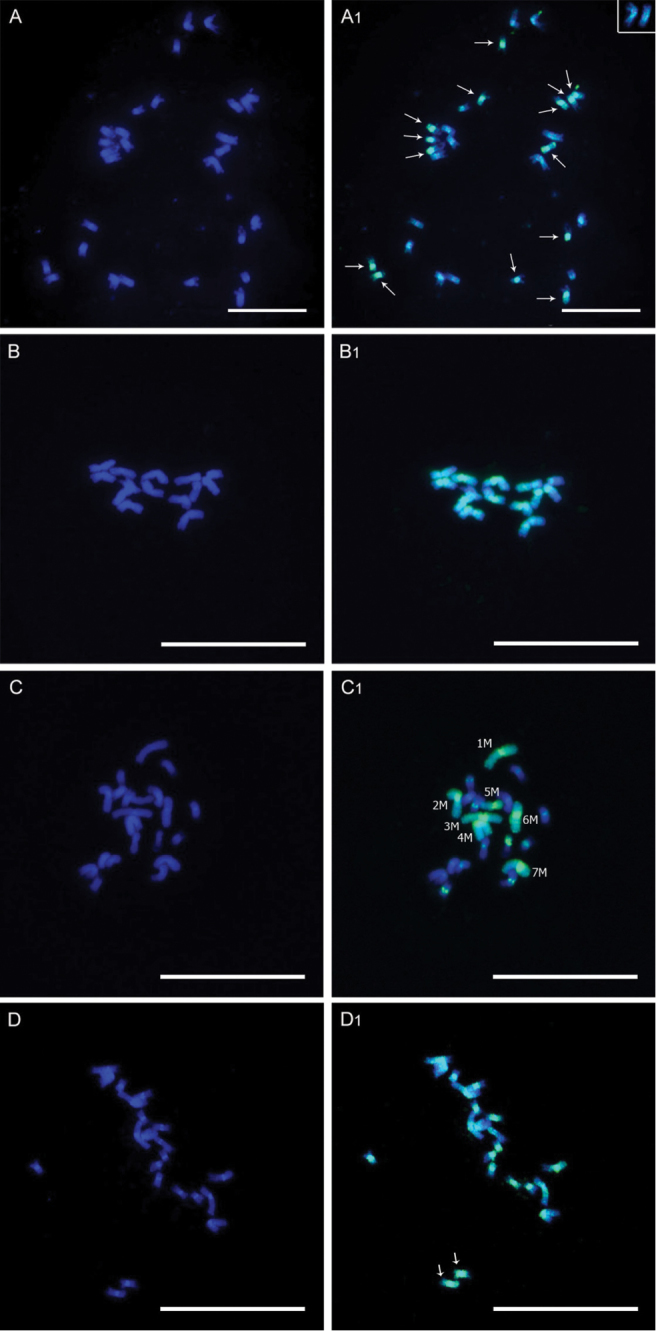
Metaphases of *Pennisetum purpureum* (**A**), *Pennisetum glaucum* (**B**), and triploid hybrid (**C** and **D**). Chromosomes stained with DAPI (**A**, **B**, **C**, **D**) and probe markings in chromosomes indicated by green fluorescence (**A1**, **B1**, **C1**, **D1**). (**A1**) chromosomes of *Pennisetum purpureum* hybridized with genomic probe of *Pennisetum glaucum* (genome **A**), (**B1**) chromosome of *Pennisetum glaucum* hybridized with genomic probe of *Pennisetum purpureum* (genomes **A**'**B**), (**C1**) chromosomes of the triploid hybrid hybridized with genomic probe of *Pennisetum glaucum* (genome **A**), (**D1**) chromosomes of the triploid hybrid hybridized with genomic probe of *Pennisetum purpureum* (genomes **A**'**B**). Bar = 10 μm (**A**); Bar = 20 μm (**B**, **C** and **D**).

**Figure 2.**
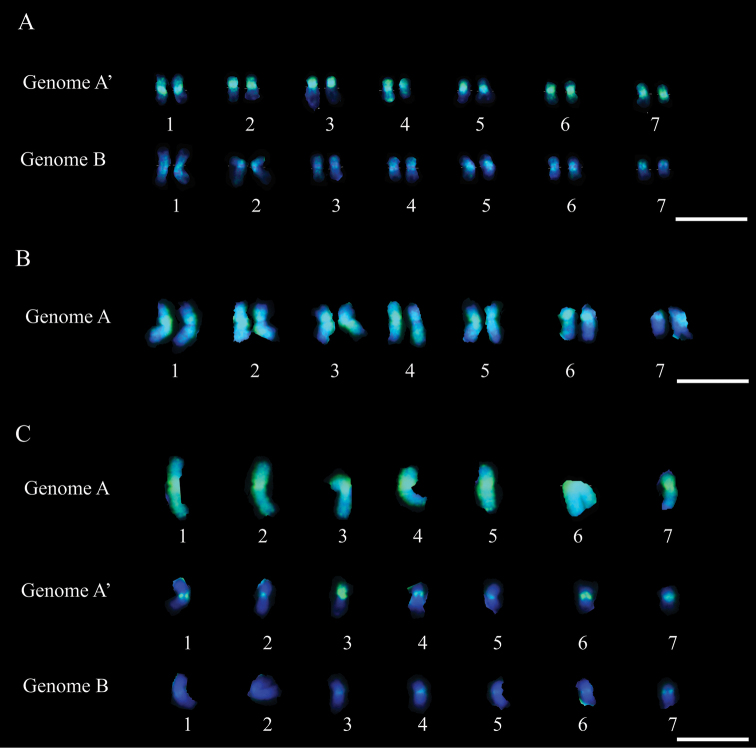
Karyograms of *Pennisetum purpureum* (**A**), *Pennisetum glaucum* (**B**) and triploid hybrid (**C**) identifying the chromosomes of genomes A, A 'and B in each genotype. Note that in (**A**) using genome A probe (*Pennisetum glaucum*), the chromosomes of genome A’ were differed from chromosomes of genome B by the staining pattern. Genome A’ chromosomes showed more apparent probe markings in green than genome B chromosomes. In (**B**), using the genome A'B probe (*Pennisetum purpureum*), all chromosomes were strongly labelled (markings in green). In (**C**), using the genome A probe (*Pennisetum glaucum*), the chromosomes of the A genome were fully labeled by the probe (markings in green), the genome A’ were strongly marked in the centromeric region and the genome B, poorly marked. It also could be note the difference in the labeling pattern between the genome A probe on the chromosomes of genome A’ in interspecific hybrid and parental *Pennisetum purpureum*. Bar = 10 μm.

The higher level of homeology between genomes A and A’ was confirmed because the 14 chromosomes belonging to genome A’ of *Pennisetum purpureum* were strongly marked and distinguished from the 14 chromosomes from genome B using the genomic DNA of *Pennisetum glaucum* (genome A) as probe in metaphases of *Pennisetum purpureum* (Fig. 1a). The chromosomes of genome A’ presented marks in along almost role chromosome length, whereas genome B presented small marks dispersed over the length of its chromosomes (Fig. 2a). Moreover, approximately 29% of *Pennisetum purpureum* genome (A’B) was hybridized by the genome A of *Pennisetum glaucum* (Table 1). This percentual represents only the A’ genome since the markers on genome B chromosomes were not record because it were dispersed on chromosomes. The observed homeology was only quantified in the genome A’ of *Pennisetum purpureum*, due to the difficulty in measuring the small and dispersed marks found in genome B (Fig. 2a).

The homeology between genomes A and A’ was confirmed by the extensive marking of *Pennisetum glaucum* chromosomes by the probe A’B of *Pennisetum purpureum*. All 14 chromosomes from genome A of *Pennisetum glaucum* were almost completely marked, with large blocks of probe signals observed on the chromosomes (Fig. 1b). The markings by the probe of genome A’B observed in the centromeric and pericentric regions represented 63% of the genome of *Pennisetum glaucum* (Table 1 and Fig. 2b). These marked portions result from hybridization, both between the genomes A and A’ and, in smaller proportion, genomes A and B, observed both in karyograms of *Pennisetum purpureum* and triploid hybrids (Fig. 2a, c).

In the triploid hybrid (AA’B) the hybridized portion of genome A (*Pennisetum glaucum*) corresponded to 54%, and the signal of probe A’B (*Pennisetum purpureum*) to 49% of its total genome (Table 1). Despite the similarity in proportion, the distribution pattern for the probes from the parental individuals was different in the hybrid (Fig. 1c). The seven chromosome of the hybrid were entirely marked with the probe of genomic DNA from *Pennisetum glaucum*. The remaining chromosomes from *Pennisetum purpureum* parental (genome A´B) presented marks only in the centromeric and pericentromeric regions (Fig. 2c). However, when the probe with DNA of *Pennisetum purpureum* was used in chromosomes of the triploid hybrid the marks were observed mainly in centromeric and pericentromeric regions, but some chromosomes appearing almost totally marked (Fig. 1d).

The differences in marking pattern observed in the triploid hybrid, mainly between genomes A and A’, could be explained by the presence of two genomes (A’ and B) in the same probe. The observations evidence the changes arising from interspecific hybridization in *Pennisetum purpureum* genomes. Once combined in a polyploid hybrid nucleus, extensive reorganization may rapidly occur in the parental diploid genomes, both intra and intergenomically (Soltis and Soltis 1999; Chen et al. 2006). The rapid intergenomic rearrangements in polyploids in relation to the diploid progenitors have been demonstrated in allohexaploid F1 hybrids of *Avena sativa* (Leitch and Bennett 1997), in hexaploids of wheat (Nelson et al. 1995) and soybean (Shoemaker 1996), as well as in triploid hybrids embryos of *Pennisetum* (Campos 2007).

Besides the existing homeology among genomes A, A’ and B, the utilization of GISH in the genomes of *Pennisetum glaucum*, *Pennisetum purpureum* and interspecific hybrid enable to verify the differences in chromosomes size, and also chromosomes number of these species. Analyzing cells of the interspecific hybrid, the difference in size between the parental chromosomes becomes evident, with those of *Pennisetum glaucum* being larger (Fig. 2c). It can also be observed that the total length of *Pennisetum purpureum* chromosomes did not increase proportionally in relation to those of *Pennisetum glaucum* (Table 1 and Fig. 2a and b), and that the chromosomes of genome B do not differ significantly in size in relation to genome A’ (Table 2 and Fig. 2a). These differences in size and chromosome number between the two species reflect their evolutionary history.

The evolutionary tendency among true grasses, which have a common and recent origin, is that the most derived species have emerged after reduction of the number and increase of the size of chromosomes in relation to the ancestors (Avdulov 1931; Bennetzen 2007; Crepet and Feldman 1991; Martel et al. 2004; Paterson et al. 2004; Stebbins 1956, 1971). This tendency applies to the genus *Pennisetum*, whose common ancestral pattern presented the basic number of chromosomes x = 9. Furthermore, the evolutionary pattern inside the genus follows the same tendency, this way, it is observed that the ancestor of the species from section *Pennicillaria*, to which *Pennisetum purpureum* and *Pennisetum glaucum* belong, presented basic number of chromosomes x = 7. In this sense, analyzing the phylogeny of the genus *Pennisetum* presented by Martel et al. (2004) and the information on chromosome size and homeology of genomes A, A’ and B observed in the present work, it can be inferred that the species *Pennisetum purpureum* and *Pennisetum glaucum* have concomitantly diverged from the common ancestor. The origin of *Pennisetum purpureum* occurred at the interspecific hybridization event, combining the genome A of the ancestor with genome B of a second, still unknown. Therefore, genome A’ could be considered a subgenome of the ancestor A due to genomic and structural changes that occurred during evolution. On the other hand, the species from the primary genic pool of *Pennisetum glaucum* have diverged from the common ancestor through increase of chromosome size, probably by increment in the genic sequences. The increase in chromosome size in this species could be explained, as described by Poncet et al. (2002), by duplication of some genes as consequence of domestication syndrome. The presence of non-homologous recombination observed among the chromosomes of *Pennisetum glaucum* reinforces the hypothesis of genic duplication as part of the differentiation in this species (Jauhar 1970; Martel et al. 2004).

This hypothesis, presented for the evolution and divergence of *Pennisetum purpureum* and *Pennisetum glaucum* from the common ancestor, may be further reinforced by the differences observed in relation to the size of chromosomes from genomes A, A’ and B, as shown in Table 2. Analyzing the size of the monoploid complement of genome A, it can be verified that it is 24% larger in relation to the length of the chromosomes of genome A’ of *Pennisetum purpureum*. Considering that the genomes A and A’ have evolved from an ancestor genome A, the difference in chromosome size could be related to genic duplication in *Pennisetum glaucum* and to genomic rearrangements observed in the allotetraploid hybrid *Pennisetum purpureum*. Rearrangements and loss of genomic sequences are common events after hybridization (Kellis et al. 2004), as observed in this study by comparison between different genomes combined in the triploid hybrid (Table 2). In this case, a reduction of 60% of genome A can be observed in the hybrid in relation to the parental *Pennisetum glaucum*, along with 44% and 52% reduction of genome A’ and B, respectively, in relation to the parental *Pennisetum purpureum*.

In this work, GISH confirmed the homeology among genomes A, A’ and B and enabled the identification and distribution of the homeologous regions in the chromosomes. Moreover, the GISH markings were able to separate the different genomes, leading the comparison on the size of the chromosomes in each of these three genomes. This distinction of the different genomes confirmed the occurrence of rearrangements after interspecific hybridization, especially when the synthetic triploid hybrid was analyzed, and prove the allotetraploid origin of *Pennisetum purpureum* It also show that genomes A’ and B have chromosomes similar in size. In evolutionary terms, the results reinforce that the genomes A and A’ have diverged from an ancestral genome A by increase of chromosome size in *Pennisetum glaucum* and rearrangements and/or deletions in *Pennisetum purpureum*. The reorganizations occurring in the ancestral genome A during evolution have generated the subgenome A’ of *Pennisetum purpureum*.

**Table 1.** Proportion of markings of genomic probes (A and A’B) on chromosomes of *Pennisetum purpureum*, *Pennisetum glaucum* and triploid hybrid.

Genotype	Total length of the chromosomes	Total length of the probe *Pennisetum glaucum* (A)	Total length of the probe *Pennisetum pupureum* (A’B)
*Pennisetum purpureum*	64,41	18,42 (28,60%)*	-
*Pennisetum glaucum*	59,01	-	37,29 (63,19%)
Triploid hybrid	73,95	40,06 (54,19%)	36,32 (49,13%)

* Proportion occupied by the probe for each genotype

**Table 2.** Total length (µm) of monoploid complement in genomes A, A' and B for each genotype.

Genotype	Total length of the genome A	Total length of the genome A'	Total length of the genome B
*Pennisetum purpureum*	-	23,8	23,95
*Pennisetum glaucum*	29,51	-	-
Triploid hybrid	17,63	10,54	12,44
